# The sign of exploration during reward-based motor learning is not independent from trial to trial

**DOI:** 10.1007/s00221-025-07074-z

**Published:** 2025-04-15

**Authors:** Katinka van der Kooij, Jeroen B. J. Smeets, Nina M. van Mastrigt, Bernadette C. M. van Wijk

**Affiliations:** 1 Department of Human Movement Sciences, Vrije Universiteit Amsterdam, van der Boechorststraat 9, 1081BT, Amsterdam, The Netherlands; 2 Department of Psychology, Justus-Liebig-Universität Gießen, Gießen, Germany; 3 Department of Neurology, Amsterdam Neuroscience, Amsterdam University Medical Centers, University of Amsterdam, Amsterdam, The Netherlands

**Keywords:** Exploration, Reward, Motor learning, Reinforcement learning

## Abstract

Humans can learn various motor tasks based on binary reward feedback on whether a movement attempt was successful or not. Such ‘reward-based motor learning’ relies on exploiting successful motor commands and exploring different motor commands following failure. Most computational models of reward-based motor learning have formalized exploration as a random process, in which on each trial a random draw is taken from a normal distribution centred on zero. Whether human motor exploration is indeed random from trial to trial has not been tested yet. Here we tested in a force production task whether human motor exploration is random. To this end, we compared the proportion trial-to-trial force changes in the behavioural data that have the same sign to the proportion expected in random exploration. One group of participants practiced with an adaptive reward criterion, which keeps rewarded performance close to current performance, and the other group practiced with a fixed reward criterion in which current performance can be far from reward performance. In both groups, we found a proportion same-sign changes larger than predicted. In the Adaptive group, both the learning and proportion same-sign changes were consistent with model simulations for low values of random exploration, whereas in the Fixed group both the learning and proportion same-sign changes were inconsistent with model simulations based on random exploration. This suggests that some form of non-random motor exploration contributes to reward-based motor learning.

**Supplementary Information:**

The online version contains supplementary material available at 10.1007/s00221-025-07074-z.

## Introduction

Humans can learn one-dimensional motor tasks, such as reaching in one direction, based on binary reward feedback about success and failure provided for instance with scored points for success and absence of scored points for failure. Such ‘reward-based motor learning’ is a form of reinforcement learning that relies on the exploitation of successful motor commands and exploration of other motor commands following failure. For instance, with binary reward feedback humans can learn to adapt reach direction (e.g. Izawa and Shadmehr 2011; Therrien et al. 2016), can learn to produce a certain force (van der Kooij et al. 2023), and can learn to reach with a certain curvature and direction (Chen et al. 2017; Dam et al. 2013). As exploration underlies reward-based motor learning, understanding the mechanisms by which humans learn to explore is important for understanding motor development (Lee et al. 2022) and for designing adequate paradigms for rehabilitation (Krakauer and Cortes 2018) and teaching (Bonawitz et al. 2011; Schaik et al. 2020). However, current understanding of exploration in reward-based motor learning is based on a key assumption that has not been challenged yet.

**Fig. 1:**
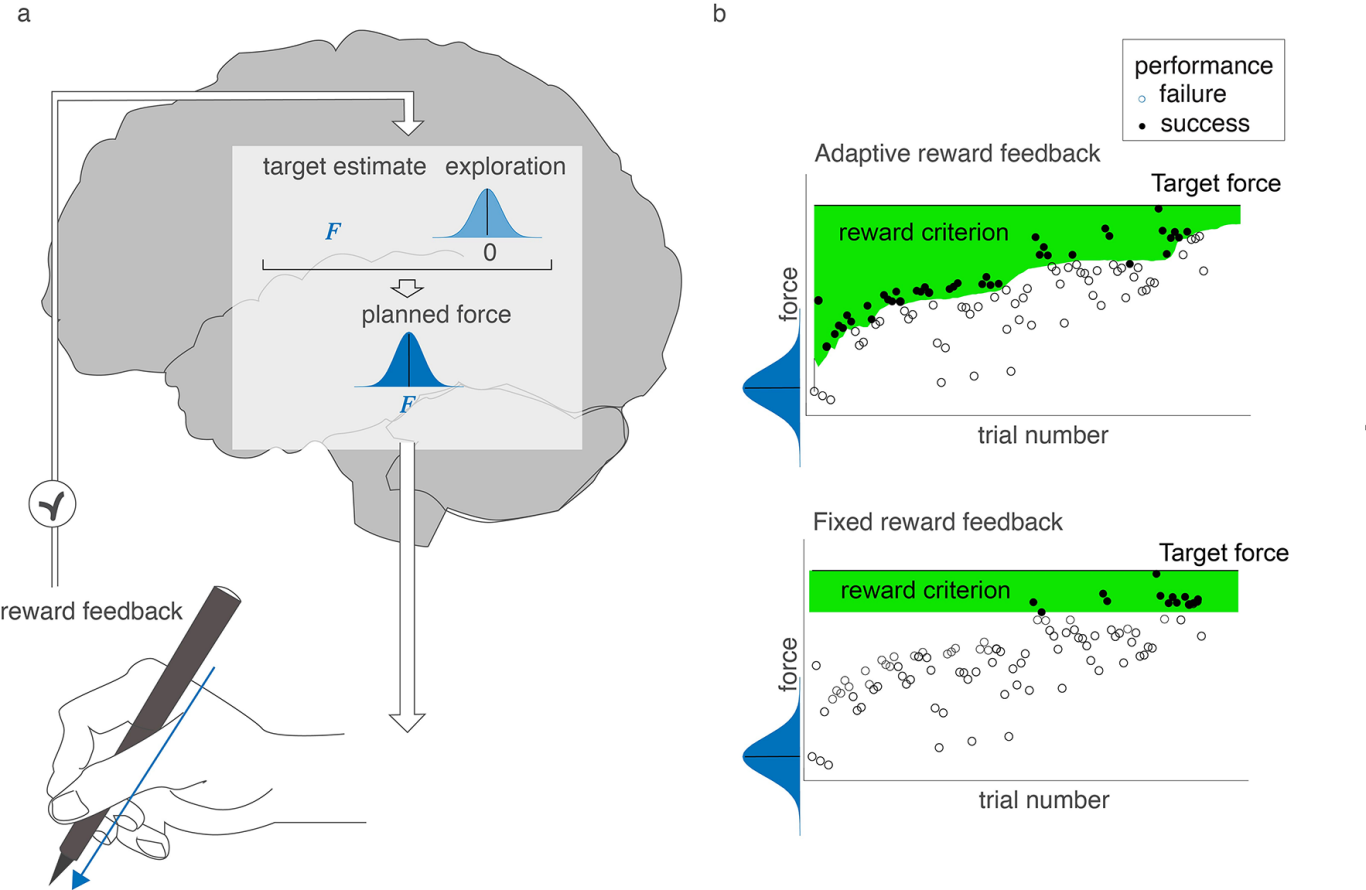
Rationale. **a**) In current models of reward-based motor learning, the planned force is a combination of a target estimate (in this case a to-be-applied force *F*) and exploration (a deviation of the target estimate that is randomly sampled from a normal distribution centered on zero). The target estimate and the amplitude of exploration are updated based on reward feedback. **b**) Illustrations of the two reward criteria. The adaptive reward criterion depends on performance (top panel), whereas the fixed reward criterion is independent of performance (bottom panel)

A key assumption underlying current computational models of reward-based motor learning is that exploration is a stochastic process in which exploration is added to a target estimate (Fig. 1). The exploration on a trial is randomly drawn from a normal distribution with a certain variability centered on zero and is thus independent across trials (e.g. Cashaback et al. 2019; Dhawale et al. 2019; Izawa and Shadmehr 2011; Sutton and Barto 2018; Therrien et al. 2018). We refer to this formalization of exploration, which may also be called ‘independent, identically distributed samples’ (Castillo et al. 2024) as ‘random exploration’. While several studies have assessed how the amplitude of exploration is controlled (Dhawale et al. 2019; Roth et al. 2023; Therrien et al. 2018), the assumption of random exploration remains to be tested.

If exploration deviates from random exploration, e.g. by showing interdependence between trials, this has implications for the quantification of exploration, which is currently based on the amplitude of variability (Pekny et al. 2015; van Mastrigt et al. 2021) or on model fits with random exploration (Hill et al. 2024; Therrien et al. 2016). If exploration involves both variability and interdepence across trials, studies measuring exploration only by variability might erroneously conclude that a participant who shows little variability with high interdependence across trials explores less than a participant who shows large variability with no interdependence across trials.

There are reasons to expect that exploration deviates from random exploration. While random exploration has the potential for motor learning even when the current performance is far from rewarded performance, learning might be unreliable and slow because extreme values are rare in the normal distribution. Hence, subsequent explorations will only rarely bring performance far from the current performance. A study with rats showed that the amplitude of variability due to exploration might be scaled to a longer reward history, resulting in large explorations when reward is absent for long while keeping explorations small when reward is frequent (Dhawale et al. 2019). This mechanism might overcome some limitations of random exploration, but it has not been tested yet whether such a mechanism facilitates human learning when performance is far from rewarded performance.

The limits of random exploration might have received little attention because experimental paradigms have defined success with a criterion that allows for learning with random exploration. In daily life, the constraints posed by the environment, such as the size and position of an apple in the tree when grasping an apple, set the criterium within which a movement is successful or not. In experimental paradigms, the experimenter defines success by rewarding certain movements. To facilitate learning, most studies start by rewarding a wide range of movements and narrow this range as performance improves (Fig. 1.b). The narrowing of the reward criterion occurs either by fixed small steps (Holland et al. 2018; Izawa and Shadmehr 2011; van Mastrigt et al. 2023) or based on how quickly the learner improves (Therrien et al. 2016, 2018; van der Kooij et al. 2018; van der Kooij and Smeets 2019). The latter adaptive procedure is also called ‘closed-loop’ feedback (Therrien et al. 2016) or ‘shaping’ (Skinner 1974). Consistent with the idea that random exploration suffices for learning when the current performance is close to being rewarded but not when current performance is further from being rewarded, such an adaptive reward criterion has been found to enhance the rate of learning relative to a fixed (‘open loop’) criterion (Therrien et al. 2016).

It is not unlikely that human exploration deviates from random exploration. First, variability in motor and cognitive tasks shows autocorrelation in which subsequent actions positively depend on each other (Castillo et al. 2024; van Beers et al. 2013). However, humans explore especially following failure (Pekny et al. 2015; Therrien et al. 2016; Uehara et al. 2019) and following failure, the autocorrelation in variability is reduced (Roth et al. 2023). Second, explicit processes contribute to reward-based motor learning (Codol et al. 2018; Holland et al. 2018; van Mastrigt et al. 2023). These explicit processes might involve strategies such as sampling exploration from range (see Supplementary S4) rather than a normal distribution, or a sweeping strategy, in which one subsequently increases the exploration in one direction and reverses this direction of exploration when the boundary of a search space is reached. Finally, exploration might be directed toward areas in the search space where most information can be gained (Wilson et al. 2021). Hence, exploration might deviate in several ways from random exploration.

The aim of the current study is to test the hypothesis that exploration during reward-based motor learning deviates from random exploration. To this end, we use a stencil-based force production task performed on a tablet (van der Kooij et al. 2023) in which we measure exploration as trial-to-trial changes in force. To assess the structure of the exploration following failure, we compare the proportion trial-to-trial changes with the same sign (either more or less force) which we refer to as ‘same-sign changes’ to the proportion expected in random exploration. We predict that the proportion same-sign changes deviates from what is expected based on random exploration.

## Methods

### Participants

In total 61 participants participated in the study. They were all students at the Faculty of Behavioural and Movement Sciences of the Vrije Universiteit Amsterdam or friends of students. The participants were divided into two groups that practiced with a different reward criterion. In the Adaptive group (*N* = 30, age 22 ± STD 3 years, 20 female, 8 male, 2 non-specified), the reward criterion was adapted to the participant’s performance whereas in the Fixed group (*N* = 31, age 21 ± STD 3 years, 23 female, 6 male, 2 non-specified), the reward criterion was fixed. The data of 6 of the 30 participants in the Adaptive group 24 were measured specifically for the current study; the other 24 were measured using a slightly different protocol in a previous study (van der Kooij et al. 2023). All participants in the Fixed group were measured in the current study. Ethical approval for both studies was provided by the local ethics committee.

### Task

**Fig. 2:**
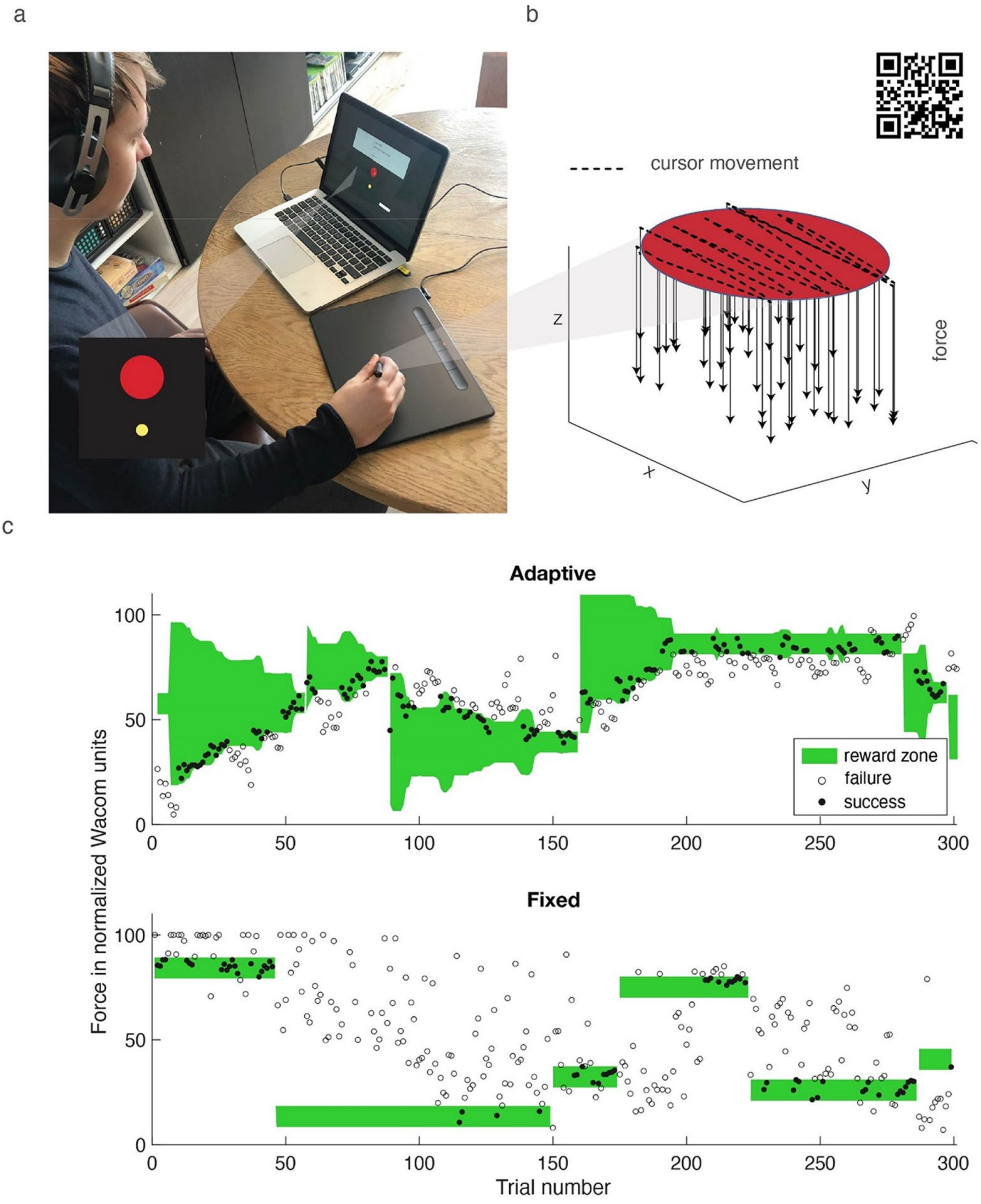
Task. **a**) Picture of a participant performing the task. The lower-left inset shows the start position (yellow) and the target circle (red) when receiving feedback on failure. **b**) Trajectory of the movement of the stylus during a single trial (dashed line) sampled at 16 ms intervals. The QR code can be scanned to view the video that was used to instruct the participants about the task. **c**) Example data and reward criterion for a 300-trial session of a participant in the Adaptive group (top row) and for a participant in the Fixed group (bottom row). Dots are the registered forces during each trial; filled dots correspond to rewarded trials. The green area corresponds to the reward criterion

The task was the same as in a previous study (van der Kooij et al. 2023), except for the addition of a fixed reward criterion and the task being administered in the lab rather than at home. Participants performed a stencil-based task in which they viewed 1 cm diameter circular targets (random colour other than red or green) at the centre of a laptop screen and made erasing-like movements on a Wacom tablet (Intuos Medium, 4096 pressure levels) positioned on the table (Fig. 2) with a stylus that could register the force on the tip of the pen up to about 50 N. The movement of the stylus was displayed on the computer screen with the standard cursor. We report forces in normalized Wacom units (ranging from 0 to 100), rather than Newtons as part of the data was collected at home without supervision, so we are not sure about the tablet settings used. The participants were instructed to move the pen in such a way that it ‘erased’ the entire target area. They were furthermore instructed to perform the erasing movement with the correct force (randomly chosen from a range of 20–80 Wacom units); they had to find this force through binary reward feedback.

To start a trial, the participant moved the cursor to a start position (diameter 0.1 cm, 1 cm below the edge of the target). Once the cursor was in the start position, the target appeared, and the participant could move toward the target and start erasing. Once the cursor hit the target, we started recording the force with which the participant made the erasing movement. After 1.5 s we provided reward feedback based on the applied force: the mean force during the last second of the erasing movement. If a trial was rewarded, we coloured the target green. Otherwise, it turned red.

The reward criterion (range of forces that were rewarded) differed between the two groups. Participants in the Fixed group were rewarded if the applied force was within 5 Wacom units from the target force (the ‘fixed criterion, Fig. 1.b’). Participants in the Adaptive group were rewarded both when the applied force was within the fixed criterion and when the applied force was within an adaptive reward criterion. This adaptive reward criterion was based on force errors: the absolute difference between the applied force and the target force. If the force error in a trial was smaller than the 40% percentile of the errors in the previous ten trials, the trial was rewarded. For the first ten trials, participants were only rewarded based on the fixed criterion. To end the trial, the participant returned the cursor to the starting position.

For both groups, we considered the target force to be found once the applied force satisfied the fixed reward criterion in eight out of ten trials. To evoke exploration throughout the 300-trial task, we used a new target force once a target was found or had been attempted 100 times. The change of target force was communicated by a new random colour for the target. The new target force was randomly chosen, but such that it differed at least 5 Wacom unit from the previous target force, so that the old and new range of force that was rewarded according to the fixed criterion did not overlap. The task lasted 20 to 30 min. Following each resolved target, the participant indicated self-reported motivation using a Quick Motivation Index (QMI, van der Kooij et al. 2019). On a slider ranging from ‘not at all’ to ‘very much,’ participants answered two questions: (1) How much did you enjoy the task until now? (2) How motivated are you to continue? Self-reported motivation was measured as the mean QMI score across targets.

### Procedure

Participants first received information on the experimental procedure and provided informed consent. Next, they were instructed on the task with an instruction video (https://youtu.be/uda3KsNrD8g). For the current data collection, participants performed the task in the lab whereas in the previous study that was conducted during the COVID-19 pandemic, participants (24 participants in the Adaptive group) performed the task at home and were instructed in a video call.

Each participant performed 300 trials in a single session and thus experienced at least three different target forces. Participants received a break midway through the experimental phase, indicated by an instruction text. After two seconds, the next target reappeared automatically. Participants were free to decide when they initiated a movement to the target.

### Data analysis

The aim of the data analysis was to quantify the exploration and learning. All data and code for the data analysis have been made publicly available at the Open Science Foundation: https://osf.io/et8qg/.

**Fig. 3:**
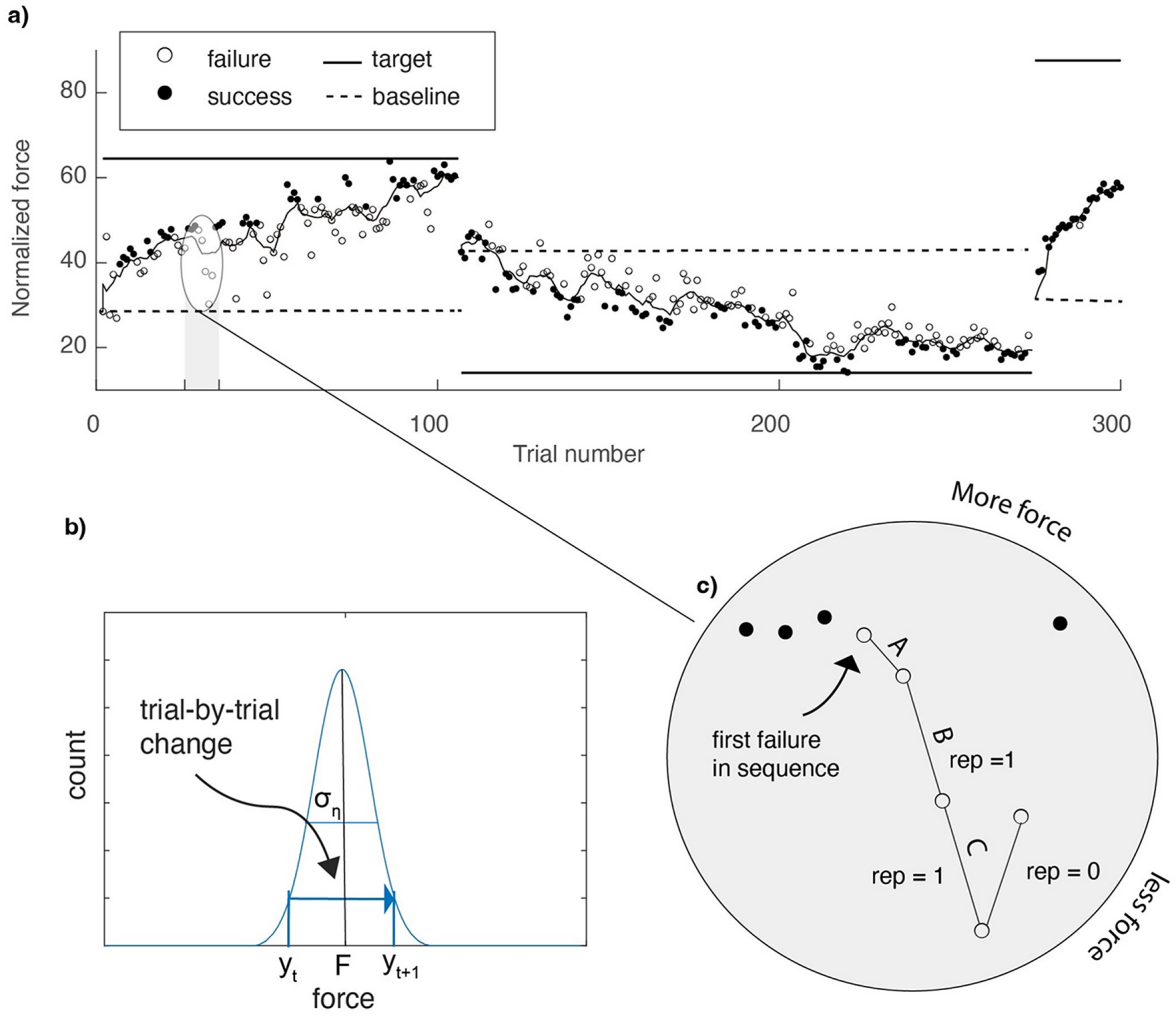
Data analysis. **a**) Forces on individual trials (circles) classified as failures and success as a function of trial number for a learner simulated with the Roth23 model. The horizontal lines indicate the target force at each trial and the curve represents the smoothed force that was used in the calculation of learning. **b**) The expected proportion same-sign changes relative to the target estimate ($\:F$), is 0.5. We measured same-sign changes as the sign of the trial-to-trial change in the applied force $\:{(y}_{t}$). **c**) Illustration of the analysis of the repetition (‘rep’) of the sign of trial-to-trial change following failure for the three exploration draws (A, B, and C) with A > B > C

To quantify the sign of exploration, we used trial-to-trial changes in the applied force after failure (Fig. 3). These were calculated as the difference in the applied force between a trial (t) and the next trial (t + 1). A same-sign change was defined as a change in the same direction (either more or less force) for two consecutive failed trials. For trials after which the target force changed and participants had to rate their motivation, we did not calculate trial-to-trial changes. The proportion same-sign changes was calculated across all failure sequences longer than one trial. The first failure did not count as an opportunity for a same-sign change as we calculated changes relative to the previous failure (Fig. 3.c).

Based on random exploration, the expected proportion same-sign explorations relative to the target estimate is 0.5. For trial-to-trial changes, the probability for same-sign changes is 1/3. We use an example to explain the underlying logic. If we draw three values for a force (A, B, C with A > B > C), these values can be presented in six orders (ABC, ACB, BAC, BCA, CAB, CBA), which are equally probable. For these six orders, only two orders (ABC and CBA) involve repetitions of the sign of trial-to-trial changes, so a probability of 1/3. This is especially true with a fixed reward criterion, which is often far from current performance and will therefore define all possible trial-by-trial changes as failures (see Fig. 1.b). An adaptive reward criterion might affect the probability for the six orders to be selected as a failure. For instance, when the target is larger than the current performance, the trial-by-trial changes in the CBA order are more likely to be classified as successes than the ABC order (see Fig. 3.c).

We are interested in the overall quality of learning and therefore used a measure that combines the rate and the amount of learning (van Mastrigt et al. 2021). To quantify learning for each target force, we first smoothed the applied force on its corresponding trials by taking a moving average with a window containing three trials before and after, hence 7 trials in total (Fig. 3.a). For the first and last three trials, the window is asymmetric, so the smoothed force on the first trial (initial error) is the average of the first four forces and the smoothed force on the last trial is the average of the last four errors. We subsequently determined the force error ($\:e$) as the absolute difference between the (smoothed) applied force and the target force. Learning of each target force was determined as the force error on the first trial (the baseline error) to a target minus the average force error on the subsequent trials to the same target force. This measure is not only larger if the final amount of learning is larger, but also if learning is faster. To obtain one measure of learning across all targets of a session, we divided the total learning across all targets by the total baseline error across all targets. With this method, a value of one represents complete learning, a value of zero represents no change in performance, and negative learning values represent a worsening of performance.

To check whether the average force difference to learn was comparable across groups, we calculated the force difference to learn as the average force error on the first trial for a target.

### Statistical analysis

We predicted that, in both groups, the proportion of same-sign changes equals 1/3 as predicted by random exploration. To test this prediction, we used a two-sided one-sample t-test on the proportion of same-sign trial-to-trial changes. To explore whether the proportion same-sign changes in the behavioural data was related to learning, we performed a Spearman rank-order correlation test on the proportion same-sign changes and the learning in each group.

As motivation has been found to depend on the reward rate (van der Kooij et al., 2021) and motivation might affect motor variability (Codol et al. 2020; Manohar et al. 2015), we compared the reward rate and self-reported motivation between groups using Mann-Whitney U rank-sum tests.

### Model simulations

We used model simulations to check whether the proportion same-sign changes following failure is indeed 1/3 in the presence of learning based on performance-dependent feedback. For this, we used four different models that have been proposed by others (Cashaback et al. 2019; Dhawale et al. 2019; Roth et al. 2023; Therrien et al. 2018). As the basic structure of the models is the same, we report the details of the earliest model (Therrien et al. 2018) here and provide the details of the other models in the Supplementary Methods.

In the Therrien2018 model, we implemented our task by defining an applied force ($$\:F$$$$\:t$$) that is the sum of a target estimate ($$\:X$$), exploration ($$\:\eta\:$$), and sensorimotor noise ($$\:m$$):$$\:{F}_{t}={X}_{t}+{\eta\:}_{t}+{m}_{t}$$

All models formalize exploration and motor noise in a trial as a random draw from a normal distribution, centred on zero, with standard deviation ($\:{\sigma\:}_{\eta\:}$ and $\:{\sigma\:}_{m}$ respectively):

$$\:{\eta\:}_{t}=N(0,\beta\:(R\left){\sigma\:}_{\eta\:}\right)$$

$$\:{m}_{t}=N(0,{\sigma\:}_{m})$$

The variability due to exploration depends on the previous success and failure and can be learned from, whereas the variability due to motor noise does not depend on feedback and cannot be learned from. The variability due to exploration ($\:{\sigma\:}_{\eta\:}$) depends on the successes and failures in the previous trial(s), with a variability scaling factor $\:\beta\:\left(R\right)$ that equals one without reward and is less than one after reward (see Supplementary Material S1 for detail)

Following reward (*R* = 1), the target estimate is updated with learning rate ($\:\alpha\:$); without reward (*R* = 0) it remains unchanged:

$$\:{X}_{t+1}={{X}_{t}+\alpha\:R}_{t}{\eta\:}_{t}$$

The models further differ in whether the target estimate is updated with the rewarded exploration only (Roth et al. 2023; Therrien et al. 2018), a combination of motor noise and exploration (Cashaback et al. 2019), or with a reward prediction error (Dhawale et al. 2019). Additionally, models can update the target estimate following reward only (Cashaback et al. 2019; Roth et al. 2023; Therrien et al. 2018) or also following failure (Dhawale et al. 2019). Crucially, all models implement exploration as a random draw from a normal distribution with a zero mean.

### Comparison of the models to the data

We simulated our experiment for each of the four models and for both reward criteria (adaptive and fixed) for 1000 learners. The simulated target force and reward feedback were determined in the same way as we did for the human participants. Also, the applied force ($\:F$) we used to determine the exploration and the learning was capped to the Wacom detection range (0–1). We assumed that our participants were not aware of this range and therefore we did not cap the target estimate ($\:X$) in the simulations.

Each learner started using an aimed random value between zero and one hundred (the full range of possible normalized forces). The free parameters in the models are the width of the distributions from which exploration ($\:{\sigma\:}_{\eta\:,R}$) and motor noise ($\:{\sigma\:}_{m}$) are drawn (Cashaback et al. 2019; Dhawale et al. 2019; Roth et al. 2023; Therrien et al. 2018), learning rate $\:\alpha\:$ (Cashaback et al. 2019; Roth et al. 2023) and the variability scaling factor ($\:\beta\:$).

As it can be assumed that the total variability is the sum of variability due to exploration and variability due to motor noise (van Mastrigt et al. 2021), we first estimated the total variability for each group ($\:{\sigma\:}_{total,group}$) from the data. For each participant, we estimated the total variability based on the trial-by-trial changes following failure as in (van Mastrigt et al. 2021). This variability was averaged per group to obtain the group total variability ($\:{\sigma\:}_{total,group}$). The standard deviation in the applied force was 5.67 for the Adaptive group and 7.86 for the Fixed group. For each of the 1000 simulated learners in the Adaptive and Fixed group, we varied the exploration fraction ($\:a$) in this variability from 0 to 1 in steps of 0.1 (Eq. 1.5).

$$\:{\sigma\:}_{\eta\:,R}=\:\sqrt{a{{\sigma\:}_{total,\:group}}^{2}}{\sigma\:}_{m,R}=\:\sqrt{(1-a){{\sigma\:}_{total,\:group}}^{2}}$$

As the learning rate does not influence exploration following failure, we based the learning rate on the values reported in the literature. The learning factor we used for the Roth2023 model was $\:\alpha\:=$0.98 (Roth et al. 2023), for the Cashaback2019 model $\:\alpha\:=$ 0.4 (Cashaback et al. 2019), and for the Dhawale model $\:\alpha\:=$ 0.23 (Dhawale et al. 2019). For the Therrien2018 model, we used a variability scaling factor $\:\left(\beta\:\right)$ of 0.2 (Therrien et al. 2018), for the Cashaback2019 and Roth2023 models we used a variability scaling factor of zero and for the Dhawale2019 model we used the variability scaling function which can be found in the Supplementary Material S1.

Additional model simulations with learning rates varying between 0 and 1 are reported in the Supplementary Material (S3). To statistically test whether the models can explain the proportion same-sign changes and learning by a certain combination of learning rate and exploration fraction, we statistically compared the median proportion same-sign changes and learning of the model simulations for each value of exploration fraction to the behavioural data using two-sided sign rank tests. We report the range of exploration fractions with which the behavioral data do not significantly differ from the simulation median.

## Results

### Behavioural data

**Fig. 4:**
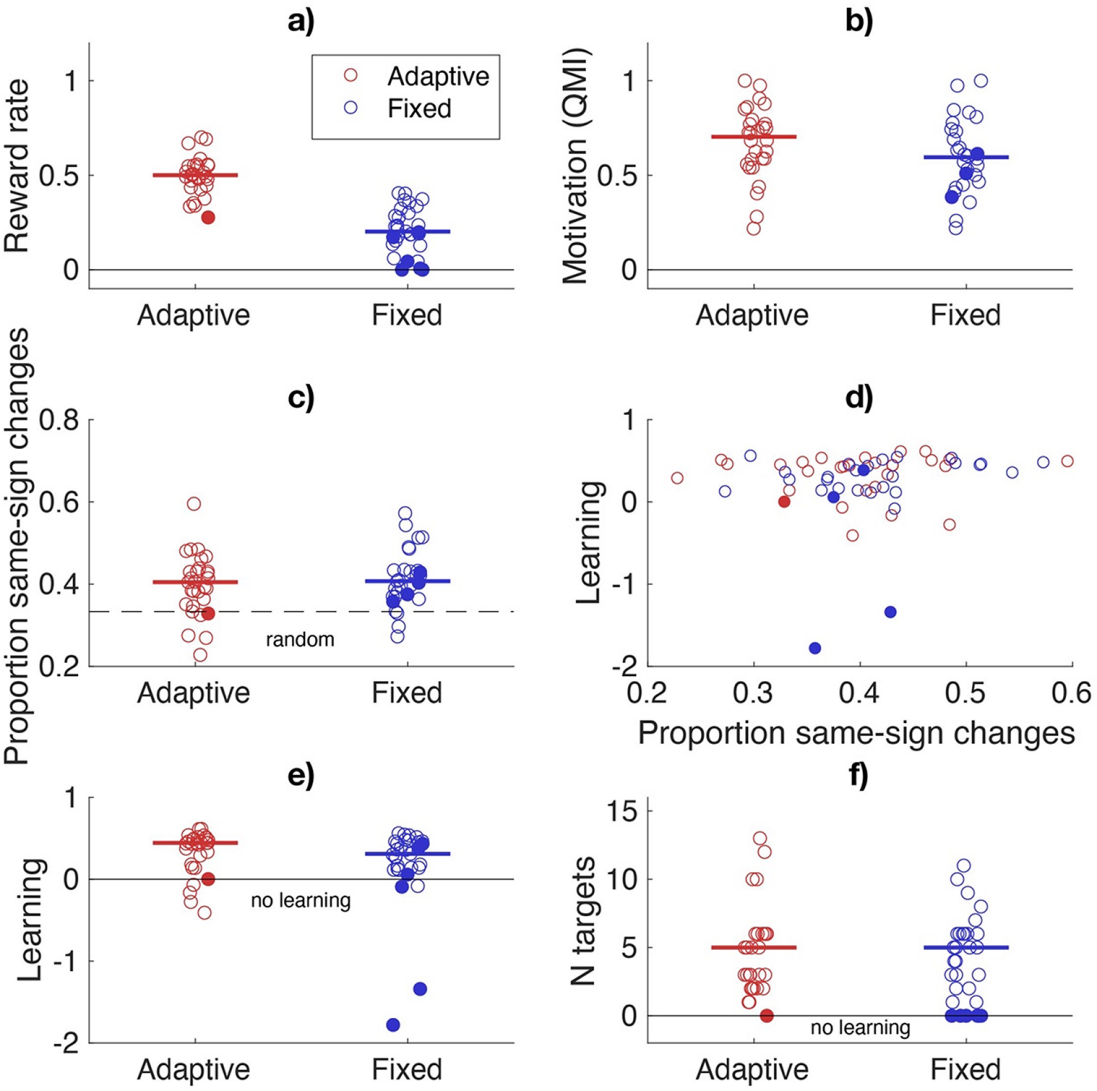
Behavioural results. **a**) The design was effective in creating a higher reward rate for the Adaptive group. **b**) The motivation did not differ between the groups. **c**) The proportion of same-sign changes was for both groups larger than in random exploration (dashed line). **d**) There was no clear correlation between learning and the proportion same-sign changes. **e**) There was no evidence that learning differed between the two groups. **f**) The number of targets resolved was similar for the two groups. Filled circles indicate participants who did not resolve any targets

As expected, the reward rate differed between the Adaptive and Fixed group (Fig. 4a). The mean reward rate for the Adaptive group was 0.50, clearly larger than the mean reward rate for the Fixed group (0.21). Despite this difference in reward rate, motivation did not significantly differ between groups (*U* = 880, *z* = 1.6, *p* = 0.12; Fig. 4b). As the target forces were randomly chosen, we also checked whether the two groups experienced comparable force differences to learn. We defined the force difference to learn as the force error on the first trial of a new target force. The mean force difference to learn ± standard deviation was 48.6 ± 17.3 for the Adaptive group and 50.9 ± 18.5 for the Fixed group. As force was measured within the Wacom detection range, we checked the percentage of trials on which the lower and upper limits of the force range were hit. In the Adaptive group, the bounds of the Wacom detection range were hit on 1% of trials and in the Fixed group, the bounds of the Wacom detection range were hit on 3.5% of the trials.

The results confirmed our prediction that in both groups the proportion same-sign trial-to-trial changes deviated from the proportion predicted by random independent exploration. The mean proportion same-sign trial-to-trial changes was higher than 1/3 in both the Adaptive group (0.40 ± SEM = 0.01, *t* = 4.58 *p* < 0.001) and in the Fixed group (0.41 ± SEM = 0.01, *t* = 6.15, *p* < 0.001) (Fig. 4c). We assessed whether the proportion same-sign changes was correlated to learning (Fig. 4d). This would especially be expected in the Fixed group where the rewarded performance could be far from the current performance and autocorrelation might be needed to hit the rewarded performance. The correlation in the Adaptive group was weak and not significantly different from zero (*rho* = 0.17, *p* = 0.39). The correlation in the Fixed group was moderate in size but not significantly different from zero (*rho* = 0.32, *p* = 0.09). These correlations did not change meaningfully when we removed three participants learning smaller than − 0.4 (see Fig. 4e).

The median learning for the Adaptive group was 0.44 and the median learning for the Fixed group was 0.33 (Fig. 4.e). The median number of targets resolved was 5 for both the Adaptive group and Fixed group (Fig. 4.f). Six participants in the Fixed group did not resolve any target whereas in the Adaptive group, only 1 participant did not resolve any targets. Figure 4 depicts these participants with filled circles and shows that they did not deviate from the other participants in the motivation (Fig. 4.b) or proportion of same-sign changes (Fig. 4.c). We exploratively compared the learning and number of targets resolved between groups with two-sided Mann-Whitney U tests and found that the both the median learning and number of targets resolved did not differ between groups (*U* = 983, *p* = 0.15; *U* = 918, *p* = 0.62 respectively). In Supplementary Material S2 we show two alternative measures of learning – the number of targets needed to resolve a target and the absolute learning - and show that these measures also do not differ between groups.

### Model simulations

**Fig. 5:**
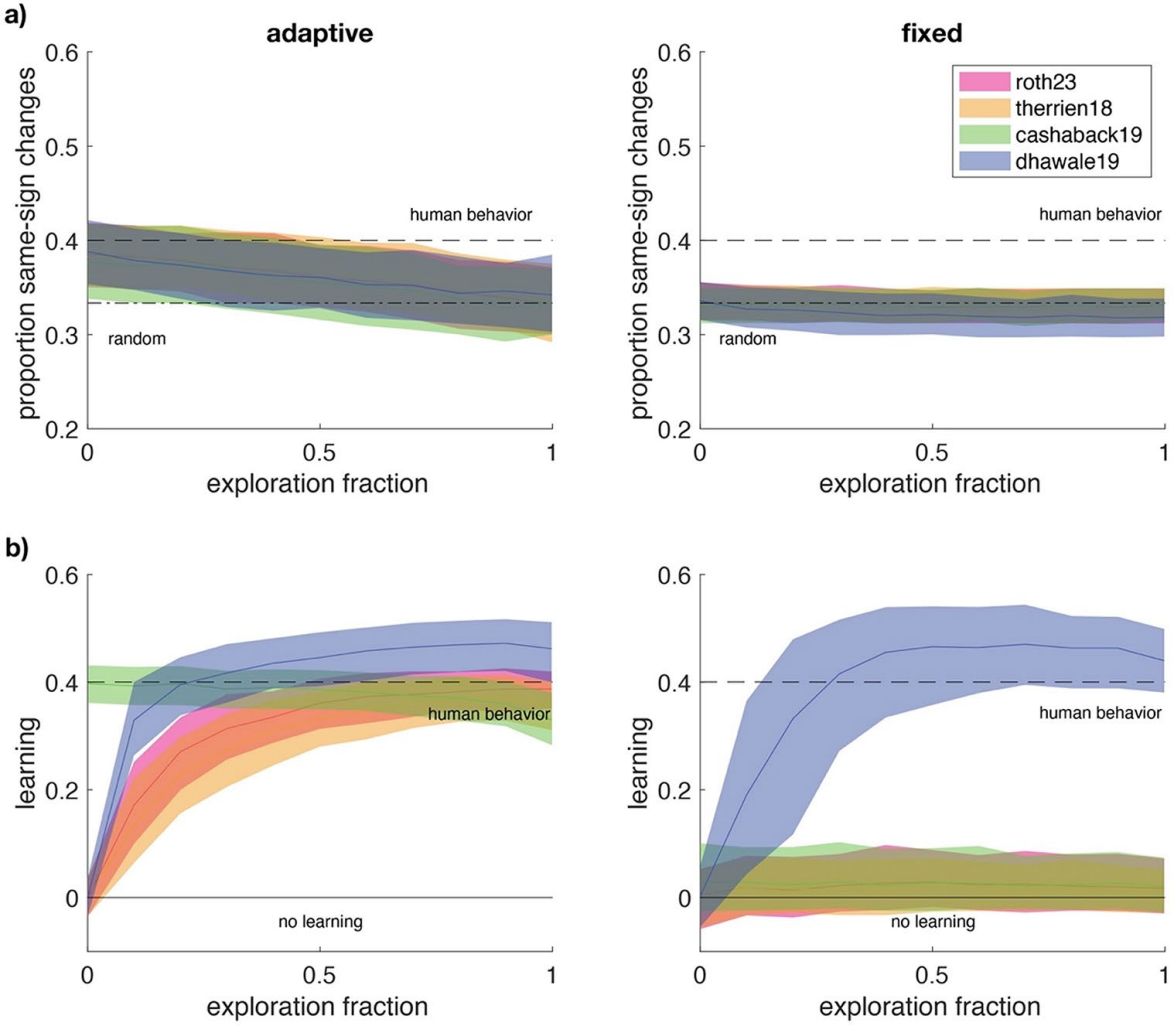
Predictions of the four models (colour) with interquartile range as a function of the exploration fraction. The horizontal dashed lines indicate human behaviour. **a**) Median proportion same-sign changes. Dashed-dotted lines indicate the prediction based on random exploration. **b**) Median learning; the horizontal lines indicate no learning

With the adaptive reward criterion (Fig. 5.a), the proportion same-sign changes in the behavioural data could be explained by relatively low exploration fractions (Roth2023: 0-0.3, Therrien18: 0-0.4, Cashaback19: 0-0.2 and Dhawale19: 0-0.2). With the fixed reward criterion, the proportion same-sign changes in behavioural data could not be explained by any fraction of exploration. The models can thus explain the human proportion same-sign changes only when an adaptive reward criterion is at play. This is because the proportion same-sign changes following failure depends not only on the exploration, but also on the classification of failure trials by the reward criterion, which tends to classify changes in the direction of the target as success and tends to classify changes in away from the target as failures. Hence certain orders of changes are more likely to be classified as consecutive failures than others (see Fig. 3). The effect of the classification of failure trials on the proportion same-sign changes is most pronounced when many of the possible trial-by-trial changes within the variability are classified as successes (see Fig. 1.a). Hence, the classification effect is strongest with the adaptive reward criterion, and low or absent with the fixed reward criterion, where the reward rate was 20%. We do not fully understand why the proportion same-sign changes reduces with the exploration fraction. Simulations with a learning rate of zero showed that the reduction in the proportion same-sign changes was not due to learning. Hence, the effect of the exploration fraction on the proportion same-sign changes must be caused by the relative increase in variability following failure.

For the learning (Fig. 5.b), we found that with the adaptive reward criterion, the models could explain learning using a broad range of exploration fractions (Roth23: 0.2-1, Therrien 18: 0.3-1, Cashaback19: 0–1, and Dhawale19: 0.1-1). With the fixed reward-criterion, only the Dhawale19 model could explain learning (exploration fractions 0.2–0.3).

## Discussion

The aim of this study was to test whether human motor exploration deviates from random exploration: taking independent random draws from a normal distribution centered on zero. To this end, we used a force-control task in which participants were rewarded according to either a fixed or adaptive reward criterion based on the force with which they moved over visual targets. Exploration was assessed by measuring the probability that trial-to-trial changes in the force would have the same sign.

The behavioural data showed a 0.4 probability of repeating the sign of exploration (either more or less force) for both reward criterion groups. Models of reward-based motor learning using random exploration (Cashaback et al. 2019; Dhawale et al. 2019; Roth et al. 2023; Therrien et al. 2018) could explain this proportion same-sign changes only for the data obtained with adaptive reward criterion and only when the amplitude of variability due to exploration was relatively low compared to the variability due to motor noise (Fig. 5.a). The models could not explain the proportion same-sign changes for the data obtained with the fixed reward criterion (Fig. 5.b). The studied models of reward-based motor learning could generate learning with the adaptive reward criterion with a wide range of values for variability due to exploration, but for the fixed reward criterion, only the Dhawale19 model could learn (Fig. 5.b), regardless of the learning fraction used (Figure S2).

The higher-than-expected proportion same-sign changes with the adaptive reward criterion might be attributed to a direction bias in the classification of failure trials by the reward criterion which tends to classify changes away from the target as failures. With the fixed reward criterion, the distance between the target and rewarded performance frequently exceeds the amplitude of variability due to exploration. Hence most trial-by-trial changes will be classified as failures and the classification bias does not apply. Due to this classification bias, the data obtained with the adaptive reward criterion are inconclusive as to whether the proportion same-sign changes with the adaptive reward criterion was caused by the reward criterion or by participant behaviour deviating from random exploration. The proportion same-sign changes in the Fixed group however shows that some form of non-random exploration contributes to human reward-based motor learning.

In the Supplementary Materials, we consider three alternative exploration strategies that might increase the proportion same-sign changes according to the models implementing random exploration for reward-based motor learning, regardless of the reward criterion used. The first alternative strategy is sampling exploration from a bounded and uniform distribution, the second strategy is autocorrelated exploration, and the third strategy is a sweeping strategy which increases the exploration in a constant direction and switches direction when the boundary of a search space is reached. Sampling from a bounded and uniform distribution did not increase the proportion same-sign changes from 1/3 to 0.4, whereas the other two strategies did result in proportions same-sign changes higher than 1/3 (Supplementary Material S4). It is unlikely however that motor exploration only involves these strategies. A problem with the autocorrelated and sweeping strategy is that when the reward criterion is adapted to performance, exploring consistently in the wrong direction causes a gradual relaxing of the reward criterion, reinforcing exploration in the wrong direction. Moreover, the sweeping strategy can bring one towards the target but when one misses the target due to motor noise, exploration drifts performance away from the target if one consistently explores in the same direction. This problem could be resolved by adapting the variability due to exploration in a more refined manner to the reward history. For instance, exploration might be directed toward areas in the search space where information can be gained (Wilson et al. 2021) or a certain number of consecutive failures should be required to initiate an exploratory sweeping bout.

Thus, while we show deviations from random exploration, we cannot pinpoint an alternative strategy that explains learning and same-sign changes in both reward criterion conditions. However, it might be wrong to search for a single strategy that explains all behaviour. Humans probably use multiple strategies in parallel and different learning strategies might be used in different learning situations. Implicit and explicit processes both contribute to reward-based motor learning (Codol et al. 2018; Holland et al. 2018; van Mastrigt et al. 2023). The implicit learning might involve random exploration whereas the explicit learning might involve directed exploration. In this case, studies focusing on implicit learning, for instance by using a double task, might observe a lower proportion same-sign changes than observed in the current study.

Measures of motor exploration have been designed to measure random exploration by focusing on the amplitude of variability (Wu and Miyamoto 2014), the amplitude of failure-induced variability (van Mastrigt et al. 2021) or estimating model parameters (Malone et al. 2023; Therrien et al. 2016, 2018). If motor exploration deviates from random exploration because trials are interdependent, measures of motor exploration shouldreflect this interdependence. This could for instanc be achieved by measuring motor exploration as a multidimensional construct which contributes to learning through both an amplitude of variability and a temporal structure containing interdependence between trials.

### Study limitations

A number of methodological choices deserves further discussion. First, the two groups differed not only in the reward criterion but also in the number of participants tested at home instead of in the lab. This might have affected motivation and measurement noise perhaps causing suboptimal learning in the Adaptive group. However, motivation in the Adaptive group was not found to be lower than in the Fixed group (Fig. 2.b). Secondly, we measured exploration and learning in a bounded task space of normalized Wacom units rather than in Newton. The upper and lower limits of the force range were only reached in 2% of the human participant trials and also in only 2% of the trials simulated with independent random exploration. Model simulations with an autocorrelated exploration strategy would frequently (18% of trials) drift beyond the boundaries of the task space however (see Supplementary S.4). Future studies assessing the structure of exploration might explicitly communicate the boundaries of a search space or use a task with a circular search space. Finally, we did not fit the models to the data as we focussed on testing a key assumption rather than testing the whole model. Future studies might compare models including interdependence across trials to current models based on random exploration by fitting them to behavioural data. A recent study showed that parameters can be reliably recovered with only a few hundred trials (Palidis and Fellows 2024).

## Conclusion

In conclusion, we show that when practicing with a fixed reward criterion, the proportion same-sign changes following failure exceeded the proportion expected based on random exploration. When practicing with an adaptive reward criterion, the proportion same-sign changes following failure could be predicted by low variability due to exploration. This was possibly due to the classification of failure trials by the adaptive reward criterion. While the learning with the fixed reward criterion can be achieved by a model implementing random exploration that scales the variability due to exploration to a longer reward history than the previous trial only (Dhawale et al. 2019), the learning with the adaptive reward criterion was achieved by all models implementing random exploration (Cashaback et al. 2019; Dhawale et al. 2019; Roth et al. 2023; Therrien et al. 2018). The results suggest that in addition to the scaling the amplitude of variability due to exploration, humans use a form of directed exploration, biased towards repeating the direction of exploration. This was especially clear when practicing with a fixed reward criterion. When practicing with an adaptive reward criterion, the proportion same-sign changes might be explained by a sampling bias in the reward criterion.

## Electronic supplementary material

Below is the link to the electronic supplementary material.

Supplementary Material 1

## Data Availability

The experimental data and the simulation code that support the findings of this study are available on the Open Science Foundation with the identifier Skinner BF.

## References

[CR1] Bonawitz E, Shafto P, Gweon H, Goodman ND, Spelke E, Schulz L (2011) The double-edged sword of pedagogy: instruction limits spontaneous exploration and discovery. Cognition 120:322–330 21216395 10.1016/j.cognition.2010.10.001 PMC3369499

[CR2] Cashaback JGA, Lao C, Palidis D, Coltman SK, McGregor HR, Gribble PL (2019) The gradient of the reinforcement landscape influences sensorimotor learning. PLoS Comput Biol 15(3):e1006839. 10.1371/journal.pcbi.1006839 30830902 10.1371/journal.pcbi.1006839 PMC6417747

[CR3] Castillo L, Leon-Villagra P, Chater N, Sanborn A (2024) Explaining the flaws in human random generation as local sampling with momentum. PLoS Comput Biol 20(1):e1011739. 10.1371/journal.pcbi.1011739 38181041 10.1371/journal.pcbi.1011739 PMC10796055

[CR4] Chen X, Mohr K, Galea JM (2017) Predicting explorative motor learning using decision-making and motor noise. PLoS Comput Biol 13(4):e1005503. 10.1371/journal.pcbi.1005503 28437451 10.1371/journal.pcbi.1005503 PMC5421818

[CR5] Codol O, Holland P, Galea JM (2018) The relationship between reinforcement and explicit control during visuomotor adaptation. Sci Rep 8(1):9121. 10.1038/s41598-018-27378-1 29904096 10.1038/s41598-018-27378-1 PMC6002524

[CR6] Codol O, Holland P, Manhor SG, Galea JM (2020) Reward-based improvements in motor control are driven by multiple error-reducing mechanisms. J Neurosci 40(18):3604–3620. 10.1523/JNEUROSCI.2646-19.2020 32234779 10.1523/JNEUROSCI.2646-19.2020 PMC7189755

[CR7] Dam G, Kording K, Wei K (2013) Credit assignment during movement reinforcement learning. PLoS ONE 8(2):e55352. 10.1371/journal.pone.0055352 23408972 10.1371/journal.pone.0055352 PMC3568147

[CR8] Dhawale AK, Miyamoto YR, Smith MA, Olvecky B (2019) Adaptive regulation of motor variability. Curr Biol 29(21):3551–3562. 10.1016/j.cub.2019.08.052 31630947 10.1016/j.cub.2019.08.052 PMC7339968

[CR9] Hill NM, Tripp HM, Wolpert DM, Maloney LA, Bastian AJ (2024) Age-dependent predictors of effective reinforcement motor learning across childhood. bioRvix, 2024.07.09, 602665. 10.1101/2024.07.09.602665 10.7554/eLife.101036 PMC12393884 40874921

[CR10] Holland P, Codol O, Galea JM (2018) Contribution of explicit processes to reinforcement-based motor learning. J Neurophysiol 119(6):2241–2255. 10.1152/jn.00901.2017 29537918 10.1152/jn.00901.2017 PMC6032115

[CR11] Izawa J, Shadmehr R (2011) Learning from sensory and reward prediction errors during motor adaptation. PLoS Comput Biol 7(3):e1002012. 10.1371/journal.pcbi.1002012 21423711 10.1371/journal.pcbi.1002012 PMC3053313

[CR12] Krakauer JW, Cortes C (2018) A non-task oriented approach based on high-dose playful movement exploration for rehabilitation of the upper limb early after stroke: a proposal. NeuroRehabilitation 43:31–40 30056438 10.3233/NRE-172411

[CR13] Lee MH, Patel P, Ranganathan R (2022) Children are suboptimal in adapting motor exploration to task dimensionality during motor learning. Neurosci Lett 770(136355). 10.1016/j.neulet.2021.136355 10.1016/j.neulet.2021.136355 34808270

[CR14] Malone LA, Hill NM, Tripp H, Wolpert DM, Bastian AJ (2023) A novel video game for remote studies of motor adaptation in children. Physiological Rep 11:e1764. 10.14814/phy2.15764 10.14814/phy2.15764 PMC10336020 37434268

[CR15] Manohar SG, Chong TTJ, Apps MAJ, Batla A, Stamelou M, Jarman P, Bhatia KP, Husain M (2015) Reward pays the cost of noise reduction in motor and cognitive control. Curr Biol 25(13):1707–1716. 10.1016/j.cub.2015.05.038 26096975 10.1016/j.cub.2015.05.038 PMC4557747

[CR16] Palidis DJ, Fellows LK (2024) Dorsomedial frontal cortex damage impairs error-based but not reinforcement-based motor learning in humans. Cereb Cortex 34:1–18 37955674 10.1093/cercor/bhad424

[CR17] Pekny SE, Izawa J, Shadmehr R (2015) Reward-dependent modulation of movement variability. J Neurosci 35(9):4015–4024. 10.1523/JNEUROSCI.3244-14.2015 25740529 10.1523/JNEUROSCI.3244-14.2015 PMC4348194

[CR18] Roth AM, Calalo JA, Lokesh R, Sullivan SR, Grill S, Keka JJ, van der Kooij K, Carter J, Cashaback JGA (2023) Reinforcement-based processes actively regulate motor exploration along redundant solution manifolds. Proceedings of the royal society B, 290. 10.1098/rspb.2023.1475 10.1098/rspb.2023.1475 PMC10581769 37848061

[CR19] Schaik JE, Slim T, Franse RK, Raijmakers MEJ (2020) Hands-on exploration of cubes’ floating and sinking benefits children’s subsequent buoyancy predictions. Front Psychol 11:1665. 10.3389/fpsyg.2020.01665 32793051 10.3389/fpsyg.2020.01665 PMC7385235

[CR20] Skinner (1974) About behaviorism. Knopf

[CR21] Sutton RS, Barto AG (2018) Reinforcement learning. The MIT Press

[CR22] Therrien AS, Wolpert DM, Bastian AJ (2016) Effective reinforcement learning following cerebellar damage requires a balance between exploration and motor noise. Brain 139(1):101–114. 10.1093/brain/awv329 26626368 10.1093/brain/awv329 PMC4949390

[CR23] Therrien AS, Wolpert DM, Bastian AJ (2018) Increasing motor noise impairs reinforcement learning in healthy individuals. eNeuro 5(3):e0050–e0018. 10.1523/ENEURO.0050-18.20181-14 10.1523/ENEURO.0050-18.2018 PMC6088368 30105298

[CR24] Uehara S, Mawase F, Therrien AS, Cherry-Allen KM, Celnik P (2019) Interactions between motor exploration and reinforcement learning. J Neurophysiol 122:797–808 31242063 10.1152/jn.00390.2018 PMC6734400

[CR25] van Beers RJ, Brenner E, Smeets JBJ (2013) Random walk of motor planning in task-irrelevant dimensions. J Neurophysiol 109:969–977. 10.1152/jn.00706.2012 23175799 10.1152/jn.00706.2012

[CR26] van der Kooij K (2021) in ’t Veld, L., & Hennink, T. Motivation as a function of success frequency. Motivation and Emotion, 45(6), 759–768. 10.1007/s11031-021-09904-3 10.1007/s11031-021-09904-3 PMC8482356 34608344

[CR28] van der Kooij K, Smeets JBJ (2019) Reward-based motor adaptation can generalize across actions. J Experimental Psychology: Learn Memory Cognition 45(1):71–81. 10.1037/xlm0000573 10.1037/xlm0000573 29698052

[CR27] van der Kooij K, Wijdenes O, Rigterink LO, Overvliet T, K. E., Smeets JBJ (2018) Reward abundance interferes with error-based learning in a visuomotor adaptation task. PLoS ONE 13(3):e0193002. 10.1371/journal.pone.0193002 29513681 10.1371/journal.pone.0193002 PMC5841744

[CR29] van der Kooij K, van Dijsseldonk R, van Veen M, Steenbrink F, De Weerd C, Overvliet KE (2019) Gamification as a sustainable source of enjoyment during balance and gait exercises. Front Psychol 10:294. 10.3389/fpsyg.2019.00294 30881322 10.3389/fpsyg.2019.00294 PMC6405433

[CR30] van der Kooij K, van Mastrigt NM, Cashaback JGA (2023) Failure induces task-irrelevant exploration during a stencil task. Exp Brain Res 241(2):677–686. 10.1007/s00221-023-06548-2 36658441 10.1007/s00221-023-06548-2 PMC9852808

[CR32] van Mastrigt NM, van der Kooij K, Smeets JBJ (2021) Pitfalls in quantifying exploration in reward-based motor learning and how to avoid them. Biol Cybern 115(4):365–382. 10.1371/journal.pone.0226789 34341885 10.1007/s00422-021-00884-8 PMC8382626

[CR31] van Mastrigt NM, Tsay JS, Wang J, Avraham G, Abram SJ, van der Kooij K, Smeets JBJ, Ivry RB (2023) Implicit reward-based motor learning. Exp Brain Res 241(9):2287–2298. 10.1007/s00221-023-06683-w 37580611 10.1007/s00221-023-06683-w PMC10471724

[CR33] Wilson RC, Bonawitz E, Costa VD, Ebitz RB (2021) Balancing exploration and exploitation with information and randomization. Curr Opin Behav Sci 38:49–56. 10.1016/j.cobeha.2020.10.001 33184605 10.1016/j.cobeha.2020.10.001 PMC7654823

[CR34] Wu HG, Miyamoto YR (2014) Temporal structure of motor variability is dynamically regulated and predicts motor learning ability. Nat Neurosci 17(2):312–321. 10.1038/nn.3616 24413700 10.1038/nn.3616 PMC4442489

