# Supplementary material for: The sign of exploration during reward-based motor learning is not independent from trial to trial
Source: Exp Brain Res. 2025 Apr 15;243(5):117. doi: 10.1007/s00221-025-07074-z (PMC12000264; doi:10.1007/s00221-025-07074-z)
Supplement: Supplementary file 1 — Supplementary Material 1 [file 221_2025_7074_MOESM1_ESM.docx]

# Supplementary material

## S1. Model equations

We used four models described in the literature to simulate the probability of same-sign changes in our task. For readability, we refer to these models by the name of the first author and the year of publication. The model equations and model parameters used to run the simulations are provided below. All models are based on equation 1.1 - 1.3 in the main manuscript which expresses the force ($F$) on a trial ($t$) as the sum of a target estimate ($X$), exploration ($\eta$), and sensorimotor noise ($m$), as (with the reward-dependent scaling factor $\beta\left( 0 \right)=1$ and $\beta\left( 1 \right)$ depending on the model):

s1

$F\left( t \right)=X_{t}+ \eta_{t}+m_{t}$; $\eta_{t}=N\left( 0,\beta(R)\sigma_{\eta} \right)$ ; $m_{t}=N(0,\sigma_{m})$

The Therrien2018 model incorporates a third source of variability, the planning noise ($p_{t}$)

s2

$$F\left( t \right)=X_{t}+ \eta_{t}+m_{t}+p_{t}$$

In all model simulations, we estimated the total variability based on the human data as in equation 1.5 in the main manuscript and assigned part of this variability to exploration based on the exploration fraction (see main manuscript, equation 1.5).

Therrien 2018 (Therrien et al., 2018):

s3

$$X_{t+1}=X_{t}+{{\alpha R}_{t}(\eta}_{t}+p_{t})$$

Roth2023 (Roth et al., 2023):

s4

$$X_{t+1}=X_{t}+{{\alpha R}_{t}\eta}_{t}$$

Cashaback2019 (Cashaback et al., 2019):

s5

$X_{t+1}=X_{t}+{{\alpha R}_{t}(\eta}_{t}$+$m_{t})$

Dhawale 2017 (Dhawale et al., 2019):

s6

$$\beta(1)={c\left( \bar{r}_{t}^{0.7}-1 \right)}^{2}$$

$$\delta_{t}={R_{t}- \bar{r}}_{t}$$

$$\bar{r}_{t}=r_{t-1}+\alpha_{r}\delta_{t-1}$$

$$X_{t+1}=X_{t}+{\alpha\delta}_{t}\eta_{t}$$

## Model parameters used in the simulations

|  | Therrien18 | Roth23 | Cashback19 | Dhawale19 |
| --- | --- | --- | --- | --- |
| $\boldsymbol{\beta(1)}$ | 0.2 | 0 | 0 | see equations s6 |
| $\boldsymbol{\alpha}$ | 1 | 1 | 0.4 | 0.2 |
| $\boldsymbol{\alpha}_{\boldsymbol{r}}$ |  |  |  | 0.4 |
| **c** |  |  |  | 6.77 |

## S.2 Alternative measures of learning

We measured learning as the area under the curve reduction which captures both the rate and the amount of learning (see Methods section). This measure involved a normalization by the total force difference to learn which could have biased the measure of learning when normalizing by small values. Based on reviewer suggestions, we analyzed two alternative measures of learning: the number of trials needed to resolve a target (Figure S1.a) and the absolute learning (Figure S1.b). The absolute learning was defined as the absolute difference between the force error on the first four trials with a target and the last four trials with a target. For both measures, we compared the learning between groups with a Mann Whitney-U rank sum test. For both measures of learning, there was no significant difference between the median of the two groups (*U* = 901, *p* = 0.41 and *U* = 905, *p* = 0.38, respectively), in line with the conclusion based on our original measure of learning.


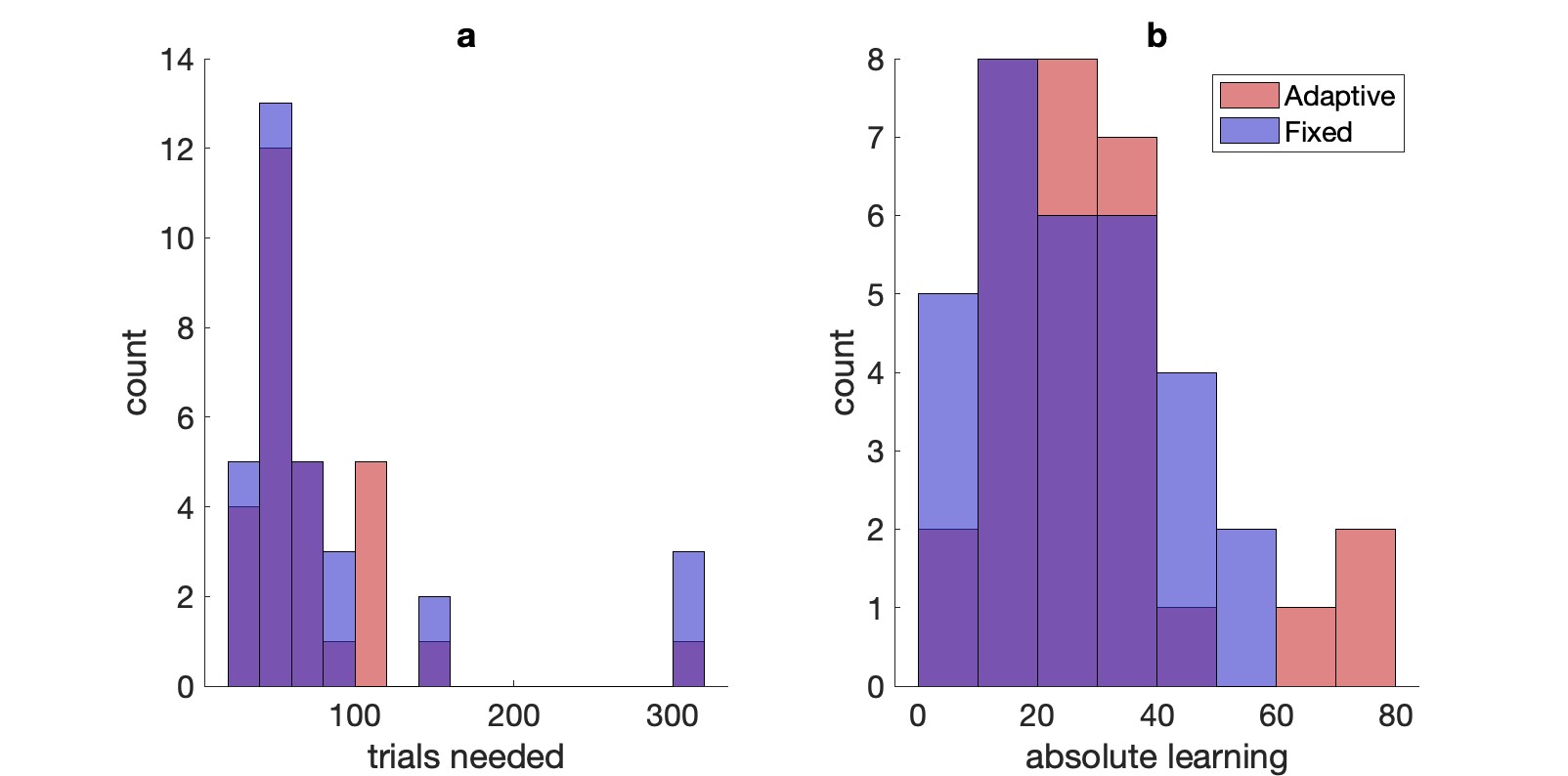


*Figure S1. Alternative measures of learning. a) Histogram of the trials needed to resolve a target for the Adaptive and Fixed group. b) Histogram of the absolute learning for the Adaptive and Fixed group.*

##

## S3. Influence of learning rate on same-sign changes and learning

Although we did not expect the learning rate to influence the proportion same-sign changes following failure or learning in the Fixed group, we ran additional simulations in which we varied for the models that include a learning rate (Cashaback et al., 2019; Dhawale et al., 2019; Roth et al., 2023) both the fraction exploration in the total variability and the learning rate from 0 to 1. For each combination of exploration and learning rate, we compared the median model simulation of the proportion same-sign changes and learning to the behavioural data using sign rank tests. Figure S2 shows that the proportion same-sign changes in the Adaptive group could be explained by some combinations of learning rate and exploration whereas the proportion same-sign changes in the Fixed group could not be explained by any combination of learning rate and exploration.


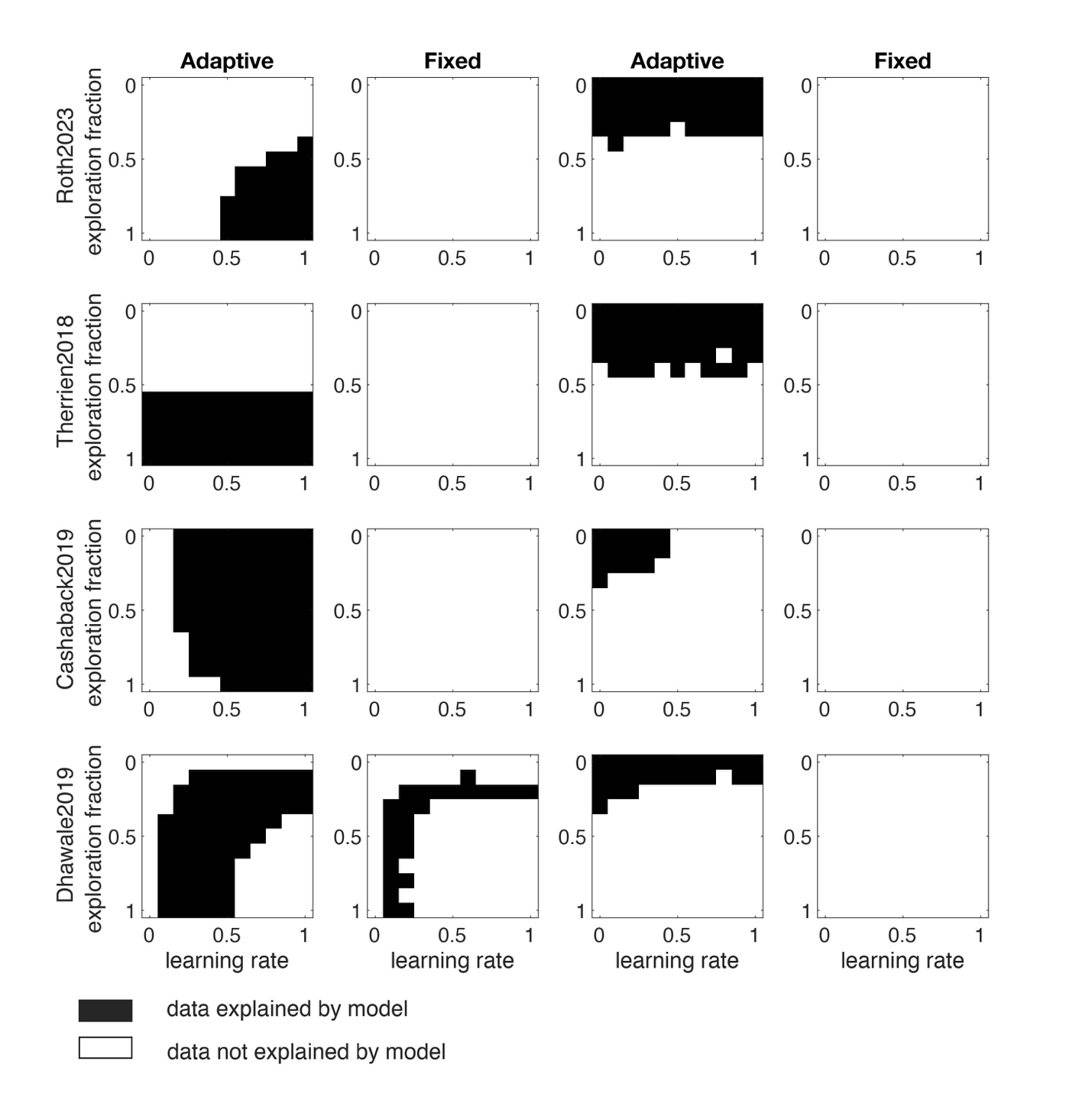


***Figure S2.*** *Influence of learning rate and fraction exploration on the congruency between the behavioural data and the model simulations with the three models involving a learning rate* (Cashaback et al., 2019; Dhawale et al., 2019; Roth et al., 2023) *of the learning and same-sign changes. Black areas indicate areas where there was no significant difference between the median model simulation and the behavioural data.*

## S4 Exploration strategies

We considered alternative exploration strategies that involved a bias towards same-sign changes and that could potentially explain the learning in both the Adaptive and Fixed group. To this end, we implemented three possible exploration strategies which we refer to as ‘bounded-uniform’, ‘autocorrelated,’ and ‘sweeping.’ The implementation of these strategies was partially based on reviewer suggestions. In the bounded-uniform strategy, the exploration was implemented as independent random draws from a uniform distribution bounded such that the sum of the target estimate ($X_{t}$) and the exploration ($\eta_{t}$) would remain within the task space (zero to one):

$$\eta_{t}=\left[ -X_{t},1-X_{t} \right]$$

In the autocorrelated strategy, the exploration was added to the previous exploration:

$$\eta_{t+1}={\beta(R)\eta}_{t}+N(0,{\beta(R)\sigma}_{\eta})$$

In the sweeping strategy, finally, the exploration was gradually increased in a constant direction until the sum of the target estimate and exploration exceeded the boundary of the task space was reached, at which moment the sign of the exploration ($a$) was reversed

$$\eta_{t}=a_{t}({\beta\sigma}_{\eta}+\eta_{t-1})$$

$$a_{t \left( X_{t}+\eta_{t} < 0 \right|X_{t}+\eta_{t}>1)}=-a_{t-1}$$

The value of $\beta$ depended on the reward as in the original models.


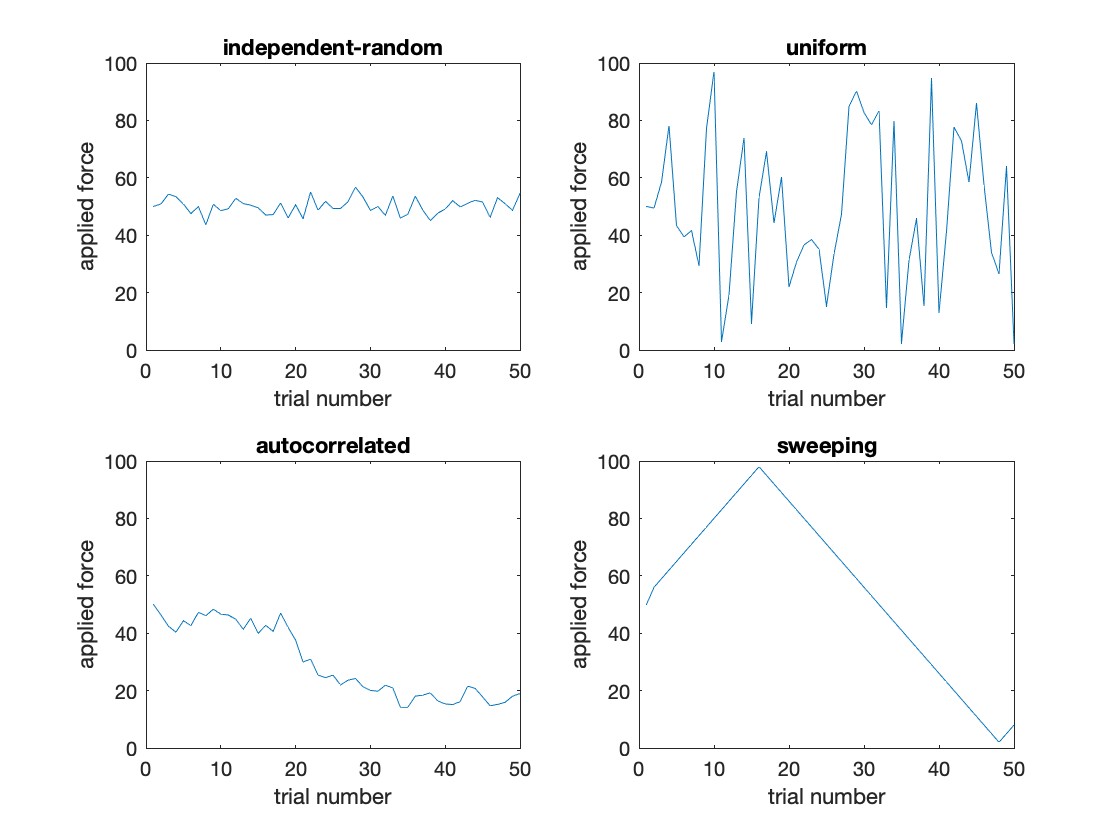


***Figure S3. Exploration strategies.*** *Illustration of how the applied force (*$F_{t}$*) would vary on 50 trials with failure feedback.*

We implemented these strategies in the Therrien18, Cashaback19 and Roth23 models, in which the amplitude of exploration is based on the previous reward only. We did not implement these strategies in the Dhawale19 model as in that model, the exploration is based on the reward history, which is already a refined exploration strategy.

For all three models, exploration strategies, and reward criteria we performed simulations in which we based the total variability on the variability in the behavioural data and varied the fraction exploration in this variability from 0 to 1 in steps of 0.1. We used the learning rates reported in the literature, which we also used in the simulations reported in the main manuscript. Figure S4 – S6 show examples of simulated learners for the three models, two reward criteria, and three strategies. In these examples, we attributed 70% of the total variance to exploration and the remaining 30% to motor noise


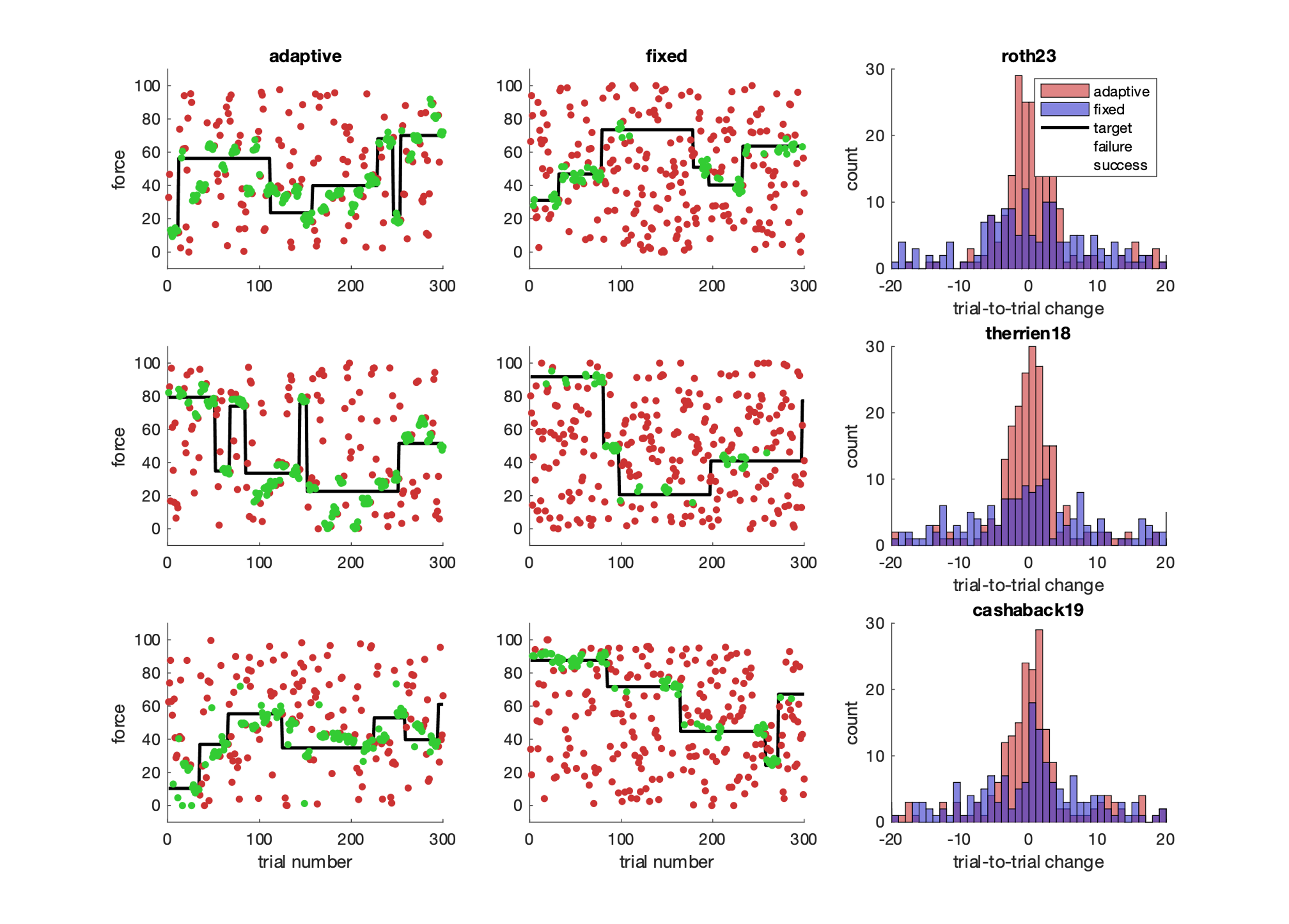


***Figure S4****. Bounded uniform strategy. Examples of simulated learners with the three models (rows) and two reward criteria (columns). The right column shows the distribution of trial-to-trial changes for the two reward criteria and three models.*

*
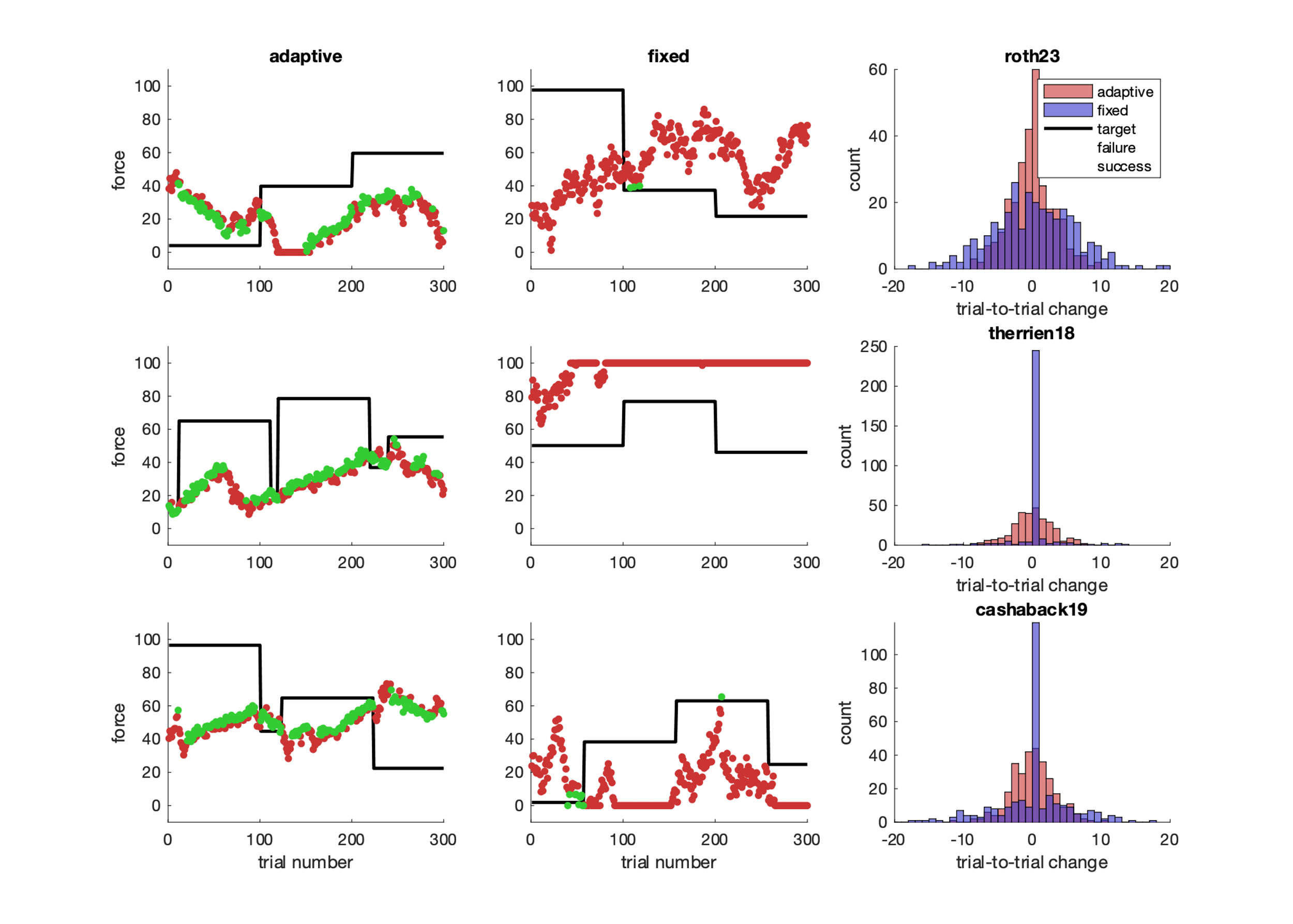
*

***Figure S5****.* ***Autocorrelated strategy****. Examples of simulated learners with the three models (rows) and two reward criteria (columns). The right column shows the distribution of trial-to-trial changes for the two reward criteria and three models.*

*
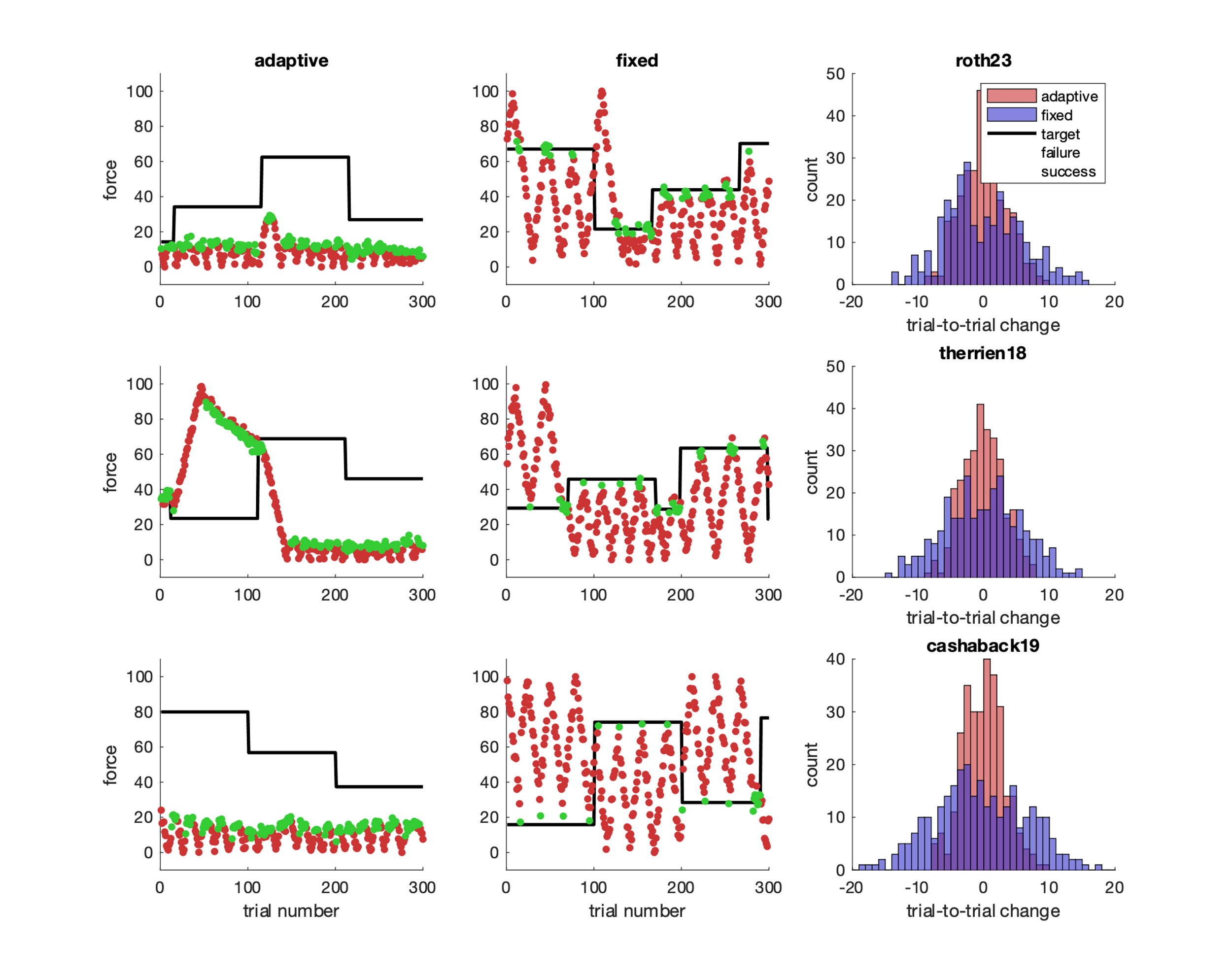
*

***Figure S6. Sweeping strategy****. Examples of simulated learners with the three models (rows) and two reward criteria (columns). The right column shows the distribution of trial-to-trial changes for the two reward criteria and three models.*

The simulations showed that although the autocorrelated and sweeping strategy could explain the proportion same-sign changes with a range of exploration fractions (Figure S7), these strategies did not explain the amount of learning with any exploration fraction as they resulted in poor learning (Figure S8). A problem with autocorrelation strategy was thatthe target estimate frequently drifted beyond the boundaries of the task space. This occurred on 19 percent of trials, in contrast to the 2% of trials on which this occurred in the behavioural data and model simulations with independent-random exploration, or with a sweeping strategy.


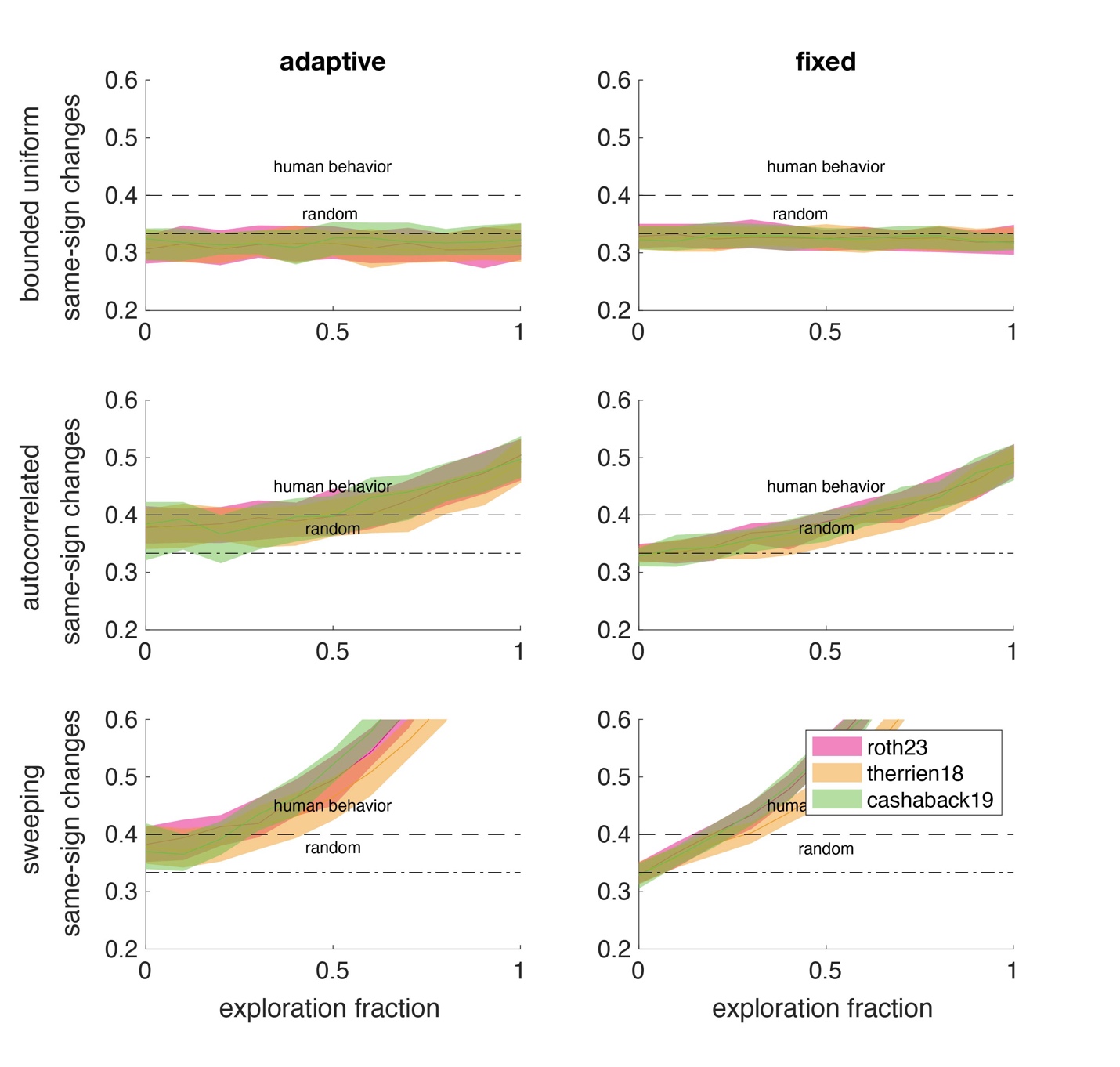


***Figure S7.*** *Simulated same-sign changes for the three exploration strategies (rows), two reward criteria (columns) and three models (colors)*

***
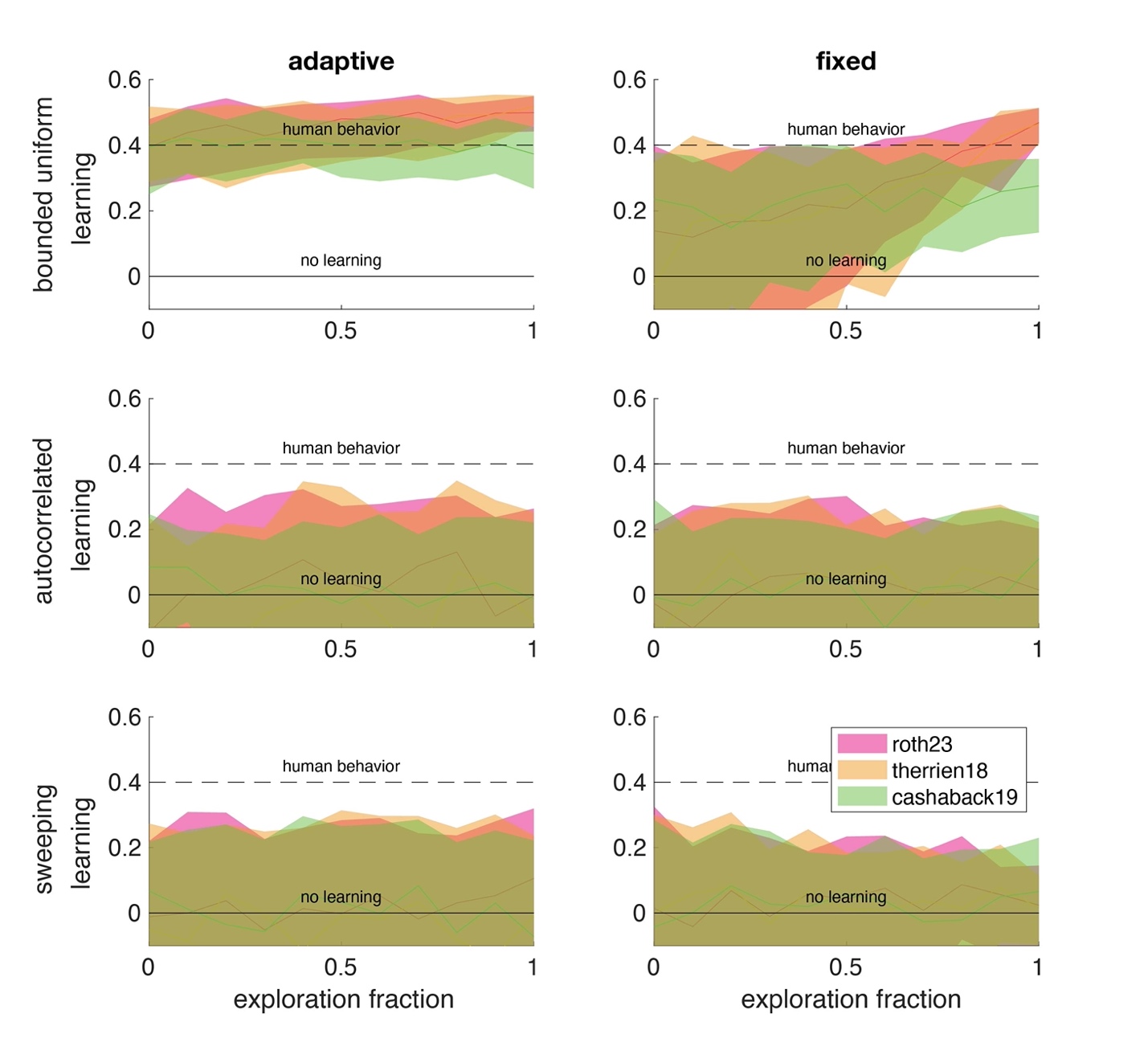
***

***Figure S8****. Simulated learning with the three exploration strategies (rows), two reward criteria (columns), and three models (colors).*

Cashaback, J. G. A., Lao, C., Palidis, D., Coltman, S. K., McGregor, H. R., & Gribble, P. L. (2019). The gradient of the reinforcement landscape influences sensorimotor learning. *PLOS computational biology*, *15*(3), e1006839. <https://doi.org/10.1371/journal.pcbi.1006839>

Dhawale, A. K., Miyamoto, Y. R., Smith, M. A., & Olvecky, B. (2019). Adaptive regulation of motor variability. *Current Biology*, *29*(21), 3551-3562. <https://doi.org/10.1016/j.cub.2019.08.052>

Roth, A. M., Calalo, J. A., Lokesh, R., Sullivan, S. R., Grill, S., Keka, J. J., van der Kooij, K., Carter, J., & Cashaback, J. G. A. (2023). Reinforcement-based processes actively regulate motor exploration along redundant solution manifolds. *Proceedings of the royal society B*, *290*. <https://doi.org/doi.org/10.1098/rspb.2023.1475>

Therrien, A. S., Wolpert, D. M., & Bastian, A. J. (2018). Increasing motor noise impairs reinforcement learning in healthy individuals. *eNeuro*, *5*(3), e0050-0018.2018 0051–0014. <https://doi.org/10.1523/ENEURO.0050-18.20181>–14
